# Rhabdomyolysis Occurring under Statins after Intense Physical Activity in a Marathon Runner

**DOI:** 10.1155/2015/721078

**Published:** 2015-02-28

**Authors:** Éric Toussirot, Fabrice Michel, Nicolas Meneveau

**Affiliations:** ^1^Clinical Investigation Center for Biotherapy, CIC-1431, FHU INCREASE, University Hospital of Besançon, 25000 Besançon, France; ^2^Department of Rheumatology, University Hospital of Besançon, 25000 Besançon, France; ^3^Department of Therapeutics and EA 4266 “Pathogens and Inflammation”, SFR FED 4234, University of Franche-Comté, 25000 Besançon, France; ^4^Department of Sport Medicine, University Hospital of Besançon, 25000 Besançon, France; ^5^Department of Cardiology, University Hospital of Besançon, 25000 Besançon, France; ^6^EA3920 “Marqueurs Pronostiques et Facteurs de Régulation des Pathologies Cardiaques et Vasculaires” SFR FED 4234, University of Franche-Comté, 25000 Besançon, France

## Abstract

Statins are widely used in the treatment of hypercholesterolemia and their side effects on muscles are well established. Conversely, data are sparse regarding the safety of this class of drugs in subjects who engage in sports, particularly those who have intense sports activity. We report the case of a marathon runner who presented with acute rhabdomyolysis during competition while being under rosuvastatin treatment. This case raises the question of the need for temporary discontinuation of statin therapy when intense physical activity is planned.

## 1. Introduction

Statins are widely prescribed for the primary or secondary prevention of cardiovascular events. It is estimated that approximately 25 million people around the world are receiving statin therapy [1]. In parallel, lifestyle counselling from our health systems is resulting in an ever-increasing number of people doing sport, sometimes at levels close to that of professional athletes. This is particularly true of running, which has become hugely popular in recent years, with more and more enthusiasts, especially competitors in long-distance races. These athletes who sometimes have dyslipidemia are usually treated. The safety of lipid-lowering drugs, especially statins, during intense physical activity in athletes is therefore increasingly topical.

## 2. Case Presentation

A 50-year old man, without any noteworthy medical history, presented with hypercholesterolemia, without cardiovascular consequences. Due to a family history of myocardial infarction and a low-density lipoprotein (LDL) cholesterol level of 1.62 g/L, atorvastatin was prescribed at a dose of 10 mg/day in November 2011 but caused severe myalgia and was rapidly replaced by rosuvastatin at a dose of 5 mg/day. The patient suffered no muscle pain under rosuvastatin. The patient was a regular long-distance and marathon runner. He was preparing for an international competition under rosuvastatin treatment without any particular side effects. On the day of the competition in April 2012 and while being still under rosuvastatin, the patient experienced progressively worsening muscular weakness. At the end of the race, he suffered from severe pains in the lower limbs similar to diffuse cramps associated with generalized muscle contraction. The pains receded about 5 minutes after rest and rehydration. Muscle enzymes (creatine kinase, CK) were tested 2 days after the race and were at 2631 IU/L (normal levels < 300) (Table 1). There was no sign of renal insufficiency or modification of thyroid-stimulating hormone (TSH) or myoglobinuria. The CK rate normalized after a few days of rest, and the rosuvastatin treatment was maintained. CK rates were tested again some time after the marathon while the patient was still under rosuvastatin and continuing normal training and were shown to be slightly elevated (Table 1). One year later, for another marathon, the patient temporarily discontinued the rosuvastatin therapy 3 months before the race. This time, the patient felt no muscle weakness at all or muscle contractions after the race. CK levels measured two days after the race were at 650 IU/L.

## 3. Discussion

This subject presented with statin-induced muscle adverse event during competition. Rhabdomyolysis is usually defined as CK level more than 10 times the upper limit of normal. In our case, CK enzymes were available 2 days after the race, at levels that were near this definition, suggesting that the subject experienced rhabdomyolysis. In addition, he had had previously severe myalgia under low-dose atorvastatin (10 mg) without marathon running, suggesting that he was a statin-susceptible person. The adverse effects of statins on muscle performance are well established [1, 2]. The frequency of muscular effects under statin therapy is estimated to range between 5 and 20% in case series or observational studies and between 1.5 and 5% in randomized studies [1]. In the PRIMO observational study of 7924 dyslipidemic patients receiving a statin in France, the frequency of muscular symptoms was 10.5% [3]. The muscular effects of statins are variable and can manifest as an isolated elevation of CK levels without clinical symptoms or may be felt as myalgia, myositis or rhabdomyolysis, or immune-mediated necrotizing myopathy [1, 2]. All these side effects are a serious limitation to statin prescription. Factors that facilitate the occurrence of such symptoms have been identified, including advanced age, female sex, a low body mass index, hypothyroidism, renal or hepatic insufficiency, intercurrent infection, vitamin D deficiency, or physical activity. Atorvastatin is reportedly more frequently associated with these side effects, contrary to hydrophilic statins (rosuvastatin, pravastatin) [1]. There is little data about the precipitating role of physical exercise in the development of muscle lesions in statin-taking subjects. The effect of statins on muscle strength and exercise performance was evaluated in a series of 420 healthy subjects who were taking either a statin or placebo. Muscle strength did not change under statin therapy, but a significant increase in muscle enzymes (CK) was observed with statin therapy versus placebo [4].

Another study reported muscle pain and hyperleukocytosis in a long-distance runner taking statins, with biological results linking the myalgia to muscle inflammation [5]. It has also been reported that competitive athletes poorly tolerate statins. In a series of 22 athletes with hypercholesterolemia, initiation of statin therapy was accompanied by onset of muscle pain in 78% of cases, regardless of the drug used or the sport [6]. Conversely, the intense physical activity associated with running a marathon (without statin therapy) can induce histological lesions corresponding to muscle fiber necrosis and inflammation [7]. Taken together, these data suggest that intense physical activity, as performed by statin-treated athletes (whether professional or not and particularly during long-distance races) could have adverse consequences on muscles. Caution is therefore required when prescribing statins in this population, or in dyslipidemic patients who may change their lifestyle and take up intense physical exercise. In these circumstances and particularly in the case of competition requiring predictably intense exercise as in our case report, a prudent approach should be preferred, recommending temporary discontinuation of statin therapy, with reintroduction of therapy after the competition.

## Figures and Tables

**Table 1:** Muscle enzyme (creatine kinase, CK) levels during training under rosuvastatin, during a marathon under rosuvastatin, and during a second marathon without rosuvastatin.

CK measurement	CK (UI/L) (normal < 300)

Marathon under rosuvastatin therapy	2631

Usual training under rosuvastatin therapy	343

Marathon without rosuvastatin	650
